# Diagnostic Performance of a Support Vector Machine for Dermatofluoroscopic Melanoma Recognition: The Results of the Retrospective Clinical Study on 214 Pigmented Skin Lesions

**DOI:** 10.3390/diagnostics9030103

**Published:** 2019-08-25

**Authors:** Łukasz Szyc, Uwe Hillen, Constantin Scharlach, Friederike Kauer, Claus Garbe

**Affiliations:** 1 magnosco GmbH, 12489 Berlin, Germany; 2 Department of Dermatology and Venerology, Vivantes Klinikum Neukoelln, 12351 Berlin, Germany; 3 MVZ Medicover Berlin, 10117 Berlin, Germany; 4 Department of Dermatology University Medical Center, 72074 Tuebingen, Germany

**Keywords:** malignant melanoma, dermatofluoroscopy, support vector machine, machine learning, melanin fluorescence

## Abstract

The need for diagnosing malignant melanoma in its earliest stages results in an increasing number of unnecessary excisions. Objective criteria beyond the visual inspection are needed to distinguish between benign and malignant melanocytic tumors in vivo. Fluorescence spectra collected during the prospective, multicenter observational study (“FLIMMA”) were retrospectively analyzed by the newly developed machine learning algorithm. The formalin-fixed paraffin-embedded (FFPE) tissue samples of 214 pigmented skin lesions (PSLs) from 144 patients were examined by two independent pathologists in addition to the first diagnosis from the FLIMMA study, resulting in three histopathological results per sample. The support vector machine classifier was trained on 17,918 fluorescence spectra from 49 lesions labeled as malignant (1) and benign (0) by three histopathologists. A scoring system that scales linearly with the number of the “malignant spectra” was designed to classify the lesion as malignant melanoma (score > 28) or non-melanoma (score ≤ 28). Finally, the scoring algorithm was validated on 165 lesions to ensure model prediction power and to estimate the diagnostic accuracy of dermatofluoroscopy in melanoma detection. The scoring algorithm revealed a sensitivity of 91.7% and a specificity of 83.0% in diagnosing malignant melanoma. Using additionally the image segmentation for normalization of lesions’ region of interest, a further improvement of sensitivity of 95.8% was achieved, with a corresponding specificity of 80.9%.

## 1. Introduction

The worldwide incidence of melanoma has risen rapidly over the course of the last 50 years. Although the mortality rates seem to level off among young adults since the mid-1990s, they keep increasing in middle aged and older adults [1]. While it has been estimated to represent between 2% and 10% of all cutaneous malignancies (depending on the country), melanoma accounts for the majority of skin cancer deaths [2,3].

Malignant melanoma is an aggressive malignancy that tends to metastasize beyond its primary site. If melanoma is diagnosed in its early stages, resection of the lesion is associated with favorable survival rates. Once melanoma is advanced, surgery is no longer sufficient, and the disease becomes much more difficult to treat. On the other hand, the urge of detecting melanoma as early as possible increases the risk of overdiagnosis: A diagnosis of a “cancer” that will not go on to cause symptoms or death. In fact, the issue of overdiagnosis is well known to dermatologists [4,5,6]. Moreover, some immature lesions might not yet develop the features that would guide a dermatologist or pathologist towards a correct diagnosis. Melanoma in situ is an example of a lesion, where the clinical diagnosis uncertainty is high, but also the accuracy and reproducibility of pathologists’ diagnoses remains low [7]. Especially (but not limited to) in such cases, there is a need for objective criteria for melanoma diagnosis that are not based solely on visual inspection of the mole or tissue pathology.

The natural pigments known as melanins are thought to play a role in the etiology and progression of melanoma, but many of their functions are currently not well understood. In general, the melanins are considered protective against sunlight-induced burns, DNA damage, and skin cancer. Those properties of melanins are due to a very broad absorption spectrum and free radical scavenging activity [8]. However, melanins are not always considered as favorable: Fair-skinned blondes and redheads who have a higher ratio of yellow–red pheomelanin to brown eumelanin are more than twice as likely to develop melanoma compared to individuals of darker skin and hair [9]. The possible explanation of deleterious role of pheomelanin in the skin is its ability to generate free radicals that activate carcinogenic processes. Ultraviolet A-irradiated mice do not develop melanoma if they lack melanin [10].

The complex response of melanin to sunlight is mediated via the fluorescent excited state. It has been shown that melanin fluorescence characteristics as spectral shape, quantum yield, and fluorescence lifetimes depend on local microenvironment and hence, melanin emission from the epidermal region of the human skin could reflect these complex processes initiated in the excited state [11]. Consequently, it should be possible to differentiate benign pigmented skin lesions from malignant melanoma based on fluorescence signals of melanin. But measuring the melanin fluorescence of the human skin is challenging. Among various endogenous (intrinsic, naturally occurring) fluorophores (chemical species that emit light upon excitation) in the human skin, melanin has the lowest quantum yield—defined as the ratio of the number of photons emitted to the number of photons absorbed. Therefore, conventional fluorescence spectroscopy (single-photon excitation with a continuous wave (cw) laser) of human skin in vivo has never been successfully used for melanin detection, as the signals are dominated by nicotinamide adenine dinucleotide phosphate (NAD(P)H) or flavins, skin fluorophores of greater quantum yield. However, when nanosecond laser pulses of 800 nm are focused in human epidermis, the fluorescence of melanin can be detected [12]. The method for early detection of malignant melanoma that is based on stepwise two-photon excitation of melanin is called dermatofluoroscopy. The CE-marked Class IIa medical device DermaFC (magnosco GmbH, Berlin, Germany) uses 800 nm, 1 ns pulses from a dye laser for excitation, and the fluorescence is detected with cooled charge-coupled device (CCD) camera. The region of interest (ROI) is then scanned with DermaFC point by point with a step size of 200 μm, resulting in several hundreds of fluorescence spectra, the exact number of which depends on the size of the area under examination. The method’s acceptance among the patients is high and has been estimated in the recent study of Fink et al., where 74% of respondents consider the dermatofluoroscopy trustworthy [13].

**Figure 1:**
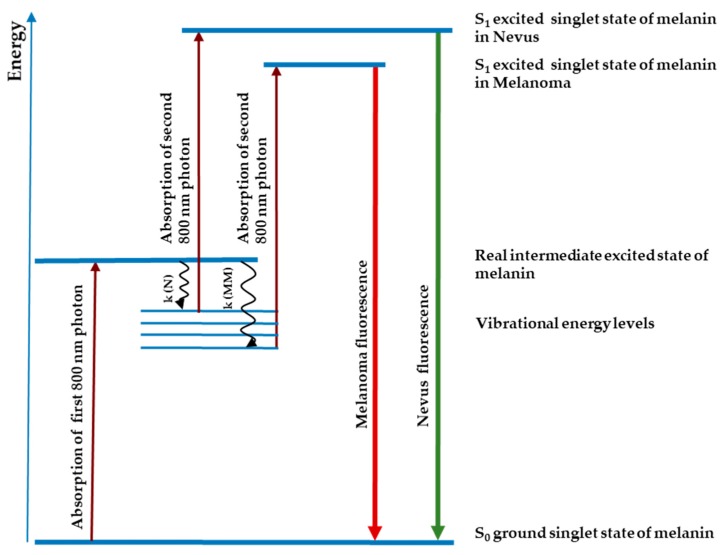
The stepwise two-photon absorption of nanosecond pulses of melanin in nevus and melanoma. The cancer-induced microenvironment alterations lead to different characteristics of the melanin first excited state, such as lifetime and non-radiative energy dissipation rates (k). The wavy lines depict non-radiative decay in nevus (N) and melanoma (MM); the colored lines show the actual bathochromic shift of melanin fluorescence in malignant melanoma compared to fluorescence of benign nevus.

Preclinical studies in freshly excised or paraffin-embedded tissue have shown that the melanin fluorescence spectra after stepwise two-photon excitation differ between cutaneous melanoma and melanocytic nevus (cf. Figure 1). Fluorescence bands peaked at around 640 nm in melanoma and at around 590 nm in melanocytic nevus. Preliminary studies on FFPE specimens revealed high sensitivity rates for the diagnosis of melanoma of 93.5% [14] and 82.0% [15]. In recent a pivotal, prospective, blinded, multicenter clinical study—FLIMMA, a total of 476 pigmented skin lesions (PSLs) were scanned with DermaFC in vivo. The sensitivity and specificity of the method was 89.1% and 44.8%, respectively [16]. The pathology served as comparator and gold standard; each tissue sample was diagnosed by one experienced dermatohistopathologist. In the FLIMMA study only PSLs clinically diagnosed as malignant melanoma or dysplastic nevus were involved. 

The aim of the retrospective study was to create a new algorithm for analysis of fluorescence data and to determine the diagnostic accuracy of the dermatofluoroscopy. The development of the machine learning algorithm was made using measurements from the FLIMMA study; however, the classification of each skin lesion as melanoma or as non-melanoma was done by three pathologists instead of one.

## 2. Materials and Methods 

**Figure 2:**
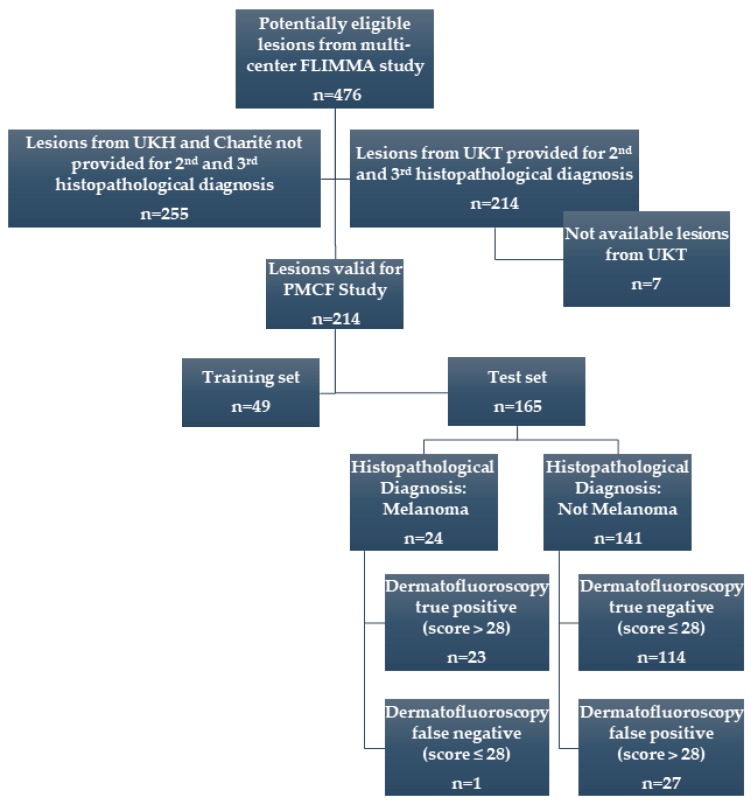
Flowchart of the post-market clinical follow-up (PMCF) study. All available samples from the FLIMMA multicenter clinical study were included, which were provided for two additional histological diagnoses.

In this paper, the results of the retrospective data analysis (post-market clinical follow-up, PMCF study) of 214 PSLs from 144 patients previously exposed to the device (in the FLIMMA study) are presented. From 476 PSLs originally involved in the FLIMMA multicenter study, 214 tissue samples from University of Tuebingen were provided for this new, post-market clinical follow-up study (Figure 2).

All available histological samples were re-examined by additional two independent dermatopathologists. The endpoint was melanoma versus non-melanoma and the consensus has been defined when ≥2 pathologists agree on a single diagnosis. In order to create and validate a new algorithm to analyze the dermatofluoroscopic data, the samples were divided into two sets: The training set of 49 lesions and the test set of 165 lesions. Based on initial (FLIMMA) pathology results, care was taken to ensure a similar proportion of the melanoma/non-melanoma samples in both sets. The diagnostic performance of dermatofluoroscopy for melanoma detection was calculated on the test set. The study was conducted in accordance with the Declaration of Helsinki and was approved by the ethics committee of the medical faculty of the University of Tuebingen, Germany.

The newly developed machine learning algorithm classifies the spectra as benign or malignant (0 and 1), whereas in the FLIMMA study the fluorescence spectra were classified into four subtypes by an artificial neural network. 

Prior to classification, the raw data (fluorescence-dominated signals measured as a function of wavelengths) collected with the DermaFC device were preprocessed. The spectral range to be analyzed was truncated to omit the signal of unknown origin of frequency above 680 nm. The spectra with low signal intensity (below 10 counts) in the range between 480 nm and 600 nm were not further considered in the data analysis. 

The spectral data were passed to the support vector machine (SVM) classifier as a 2D array (matrix) of the following dimensions: Number of observations x (number of predictors+1), where the last column is the vector of responses (here, two classes). The number of observations equals the number of the fluorescence spectra after preprocessing. The number of predictors is 750 and corresponds to the spectral range between 382 nm and 680 nm. Each spectrum in the training set is labelled according to the diagnosis of the lesion provided by pathology (supervised learning).

The solver used in this work is called an iterative single data algorithm (ISDA), and is implemented in MATLAB Statistics and Machine Learning Toolbox [17]. The most important parameters used during model optimization included: Box constraint (known also as parameter C), kernel function (such as linear, polynomial, gaussian) and kernel scale (known also as parameter σ^2^). 

To assess the predictive performance of the model (classifier) and to estimate how it performs on a new data set also known as test data, the cross-validation was used. Each round of cross-validation involved randomly partitioning the original dataset comprised of 49 samples (17,918 spectra) into a training set and a testing set. The training set was then used to train a supervised learning algorithm and the testing set was used to evaluate its performance. This process was repeated several times and the average cross-validation error was used as a performance indicator. Here, the k-fold cross validation method was used, which partitions data into k (here, 20) randomly chosen subsets (or folds) of roughly equal size. One subset was used to validate the model trained using the remaining subsets. This process was repeated *k* times such that each subset was used exactly once for validation. The final validation of how well the model predicts on new data (generalizes) was done on the set of 165 samples not implemented in the training phase of the classification model (hold-out validation). The model predicts the class (0 or 1) of each fluorescence spectrum. The lesion was classified as malignant if the score, which is a linear function of the number of ‘malignant’ spectra (class 1), exceeded the cut-off value (here, 28). 

Lastly, a correction of the final score with two factors received from image segmentation (Li thresholding as implemented in the Scikit image library [18,19,20]) was tested. Provided the lesion under investigation was not measured entirely, the final score included correction of the region of interest (ROI). The ROI defines the borders of an object under consideration; here: The boundaries of a tumor on an image. 

## 3. Results

All three pathologists agreed on the melanoma/non-melanoma diagnosis in 86% of cases (184 of 214); i.e., for 30 samples the pathological diagnosis was unequivocal. In comparison to the results of the FLIMMA-study (first diagnosis), 14 diagnoses were changed. With this improved ground truth of the data set, the classification algorithm was trained. 

The performance of three best SVM-models (trained on 49 lesions); i.e., the models with the highest accuracy and concomitant low generalization error, estimated using *k*-fold cross-validation, were finally validated using the test set of 165 samples; the data set that had been used at all in the training phase. For each PSL, the score was calculated. Table 1 shows the classification accuracy of the best SVM-Model trained using 49 dermatofluoroscopic measurements, validated on remaining 165 PSLs, with and without the correction of the ROI. 

**Figure 3:**
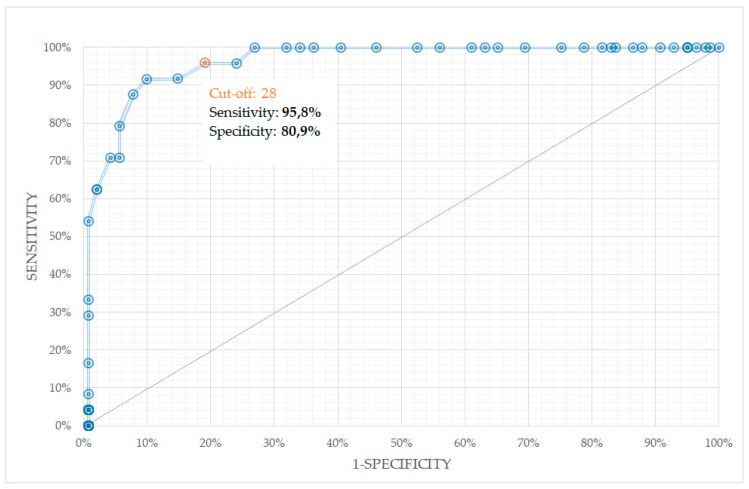
Receiver operating characteristics (ROC) curve of the best support vector machine (SVM) classifier, validated on 165 lesions, where the ROI-normalization factors were implemented in the final score calculation.

The third-order polynomial function with box constraint = 0.165 was found to be a superior kernel, with sensitivity and specificity for melanoma diagnosis 91.7% and 83.0%, respectively. When, additionally, the correction factors of ROI were implemented in final score calculation, the sensitivity increased to 95.8%, whereas the specificity decreased slightly to 80.9%, with diagnostic accuracy of 83.0% and diagnostic odd ratio (DOR) of 53.625. The corresponding receiver operating characteristics (ROC) curve is depicted in Figure 3. The area under the curve (AUC) of ROC was 0.946. The correction of ROI is of particular importance when scanning the lesion with pronounced or dominated regression (see next paragraph for more details).

**Figure 4:**
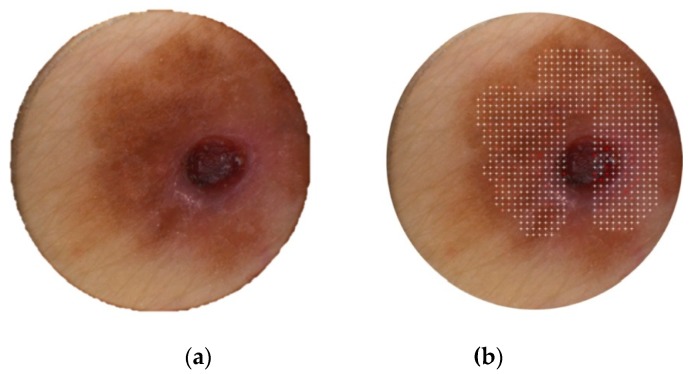
(**a**) The pigmented skin lesions (PSLs) not recognized as melanoma with dermatofluoroscopy. According to two of three pathologists, the lesion is an example of surface-spreading melanoma (SSM); (**b**) note the questionable scan area chosen.

With the cut-off value of 28, only one melanoma was overseen (see the lesion photo in Figure 4); two of three histopathologists classified the lesion as surface-spreading melanoma (one diagnosis involved tumor thickness of 0.31 mm). One pathologist diagnosed this lesion as dysplastic nevus. The DermaFC final score was 27 in this case. The scan area might not have been chosen optimally (cf. Figure 4b): the central, conspicuous part of the lesion was measured only in around 50%. 

Out of 141 non-melanoma lesions, as diagnosed by pathology (at least two pathologists provided the diagnosis: Not melanoma), 114 were classified correctly with the SVM-Model of the magnosco DermaFC device. The average final score (cut-off: 28) of the true negative results was 17.17 (median = 18). The average final score of the 27 non-melanoma lesions misclassified as malignant melanoma (false positive) was 31.22 (median = 30). The average score of lesions correctly classified as melanoma (true positive) was 37.65 (median = 38). It should be noted here, that the lesions included in the study were clinically diagnosed as dysplastic nevi or melanomas. The Table 2 summarizes the false positive results. The final scores of all lesions involved in the study were in the range between 0 and 60.

## 4. Discussion

There exists an extensive literature on the computerized diagnosis of melanocytic lesions, mostly based on methods utilizing image analysis within the context of machine learning [21,22,23,24]. Very recent studies show the automated classification algorithms may actually outmatch the performance of the average dermatologist in diagnosing melanoma and other skin cancer types [25,26]. One should be, however, very careful when comparing the diagnostic accuracy of various computer-aided methods. The most important problems to be considered are: (i) Data set size in relation to the statistical method applied, (ii) quality and characteristics of the data set, (iii) performance of the algorithm on the new data set, as well as the validation approach, (iv) ground truth quality, and (v) study design.

A deeper analysis of the results of the performance of the methods based on image processing debunk a big problem in generalization of these algorithms on unseen data. In the publication of Haenssle et al. [25], a deep learning convolutional neural networks (CNN) architecture was trained and validated using 100 dermoscopic images. The CNN ROC curve revealed a higher specificity of 82.5% when compared to dermatologists (71.3–75.7%, depending on dermatologist’s experience) at their sensitivities of 86.6% and 88.9%, respectively. However, the performance of the algorithm to diagnose melanoma validated using new data revealed much lower accuracy: At a sensitivity of 89%, the specificity was only 50%, whereas for sensitivity of 94%, the specificity was around 25%. Nevertheless, the CNN algorithm was among the top three algorithms of the 2016 International Symposium on Biomedical Imaging (ISBI) [27] challenge. The main reason for relatively poor broader generalization on the new data, according to the authors themselves, involved: (i) The bias typical to reader study (a reader study is a diagnostic accuracy study aiming to assess clinical performance of one technology versus another, on the basis of image interpretation by a group of human readers); (ii) insufficient variety of lesions; and (iii) a shortage of melanocytic lesions from other skin types and genetic backgrounds. In general, the problems (ii) and (iii) can be solved by enlarging the data set and improving its quality (e.g., lesions variety). Other challenges like the reader study bias and ground truth quality are more difficult to deal with. Finally, the fact that some (especially early) melanomas do not clinically display the characteristics for cancer morphological alterations, will most probably always limit the usage of image analysis in melanoma diagnostics. 

In this study, the data set used to validate the performance of the classification model included 165 lesions not used in any way in the training set. The label noise was significantly reduced compared to FLIMMA study as all the lesions of training and test sets were independently classified as melanoma or non-melanoma by three experienced dermatohistopathologists instead of just one. Finally, the dermatofluoroscopic data provides new and more objective criteria for diagnosing melanoma. A unique art of melanin excitation allows for measuring melanin fluorescence in vivo, which spectral shape is different for melanocytes in the healthy skin, melanocytes in nevi and melanocytes in melanoma. 

Among various machine learning methods, the support vector machines (SVMs) were chosen due to their high accuracy, ability to deal with high-dimensional and large datasets, and their flexibility in modeling diverse sources of data [22,23,28,29,30,31]. For example, in comparison with neural networks, SVMs do not require arbitrary coefficients or weights, are computationally faster, avoid the problem of local extrema (SVM optimization always ends up in a global extremum due to its convex nature), and are designed to generalize well to hitherto unseen objects.

Given the advantages of the inherent efficiency and the generalization capabilities of the SVM classifiers, the model was developed, for which 95.8% sensitivity and a specificity of 80.9% were achieved on the validation set. 

In dermatofluoroscopy, the spectra of PSLs are dominated by fluorescence signals resulting from stepwise two-photon excitation of cutaneous fluorophores with 800 nm/1 ns laser pulses. Melanins’ fluorescence is crucial to detect melanoma, as its spectral shape reflects the cancer-induced metabolic alterations in cutaneous melanocytes. To a lesser extent, signals of other naturally occurring in the skin fluorophores are considered by the classification algorithm; e.g., NAD(P)H or flavins. Beside fluorescence, the second harmonic of the 800 nm excitation light dominates the collected spectra at around 400 nm. In general, second harmonic generation (SHG) requires intense laser light passing through a material with a non-centrosymmetric molecular structure. Here, the microcrystalline structure of collagen in the dermis is responsible for the frequency-doubling of the laser light. The spectrally-resolved signal detected by the CCD camera is thus the result of various physical processes, including fluorescence, absorption (including light re-absorption by fluorophores), scattering, and generation of the second harmonic. For hypomelanotic or regressive lesions, the melanin concentration in melanocytes might be too low for safe diagnostics using dermatofluoroscopy. Therefore, lesions with dominating (over 50%) regression or amelanotic melanomas were not involved in clinical study and are not suitable lesions for dermatofluoroscopy. The clinical and dermoscopic diagnosis of regressing PSLs are particularly challenging for dermatologists anyway. Recently, Rubegni et al. proposed a score model that, with high sensitivity and specificity (97.8% and 75.5%, respectively), allows differentiation between melanomas with regression and regressing nevus [32].

**Figure 5:**
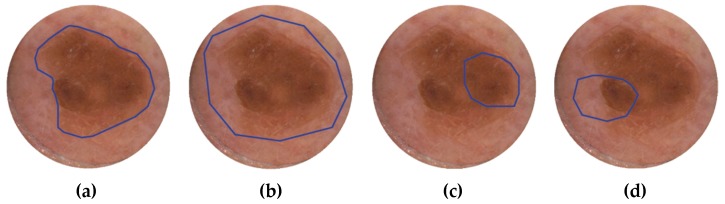
The ROI normalization principle. Four different scenarios in the clinical routine: (**a**) The region of interest (lesion boundaries) matches the area of measurement chosen by the DermaFC operator; no correction factors are used. (**b**) The whole lesion is measured together with the surrounding skin outside the tumor. (**c**) The fraction of the lesion and no healthy skin is measured. (**d**) Both factors are used for normalization of ROI, as the lesion is not completely measured, and a significant part of the scan area covers the skin around the tumor.

In the FLIMMA study, some skin lesions were scanned with DermaFC only partially, or involved less pigmented areas, such as healthy skin outside the tumor or regressive zones. In order to estimate how much measuring such areas may affect the diagnostic accuracy of DermaFC, the correction factors to the score calculated from the number of “malignant” spectra, as classified by SVM-algorithm, were introduced. A simple segmentation algorithm was used to find the lesion boundary and its area was compared to the area chosen by the operator (dermatologist). As a result, two normalization factors were introduced: One describing which fraction of the segmented lesion was scanned with the DermaFC, and the second contained the information how much the scanning area involved low-pigmented regions. In Figure 5, the ROI correction principle is demonstrated. If the ROI chosen by the medical personal matches the lesion area (recommended procedure), the final score will not be affected by these factors. Otherwise, the final score is adjusted accordingly. 

Without ROI normalization, the PMCF study reveals a lower sensitivity of 91.7%, but specificity is slightly higher when compared to the algorithm utilizing normalization factors. As the “sensitivity classifiers” are preferable in melanoma diagnostics, the automatic ROI normalization procedure is considered beneficial. It is important to note here, a relatively small impact of the ROI correction factors, on overall diagnostic accuracy of the DermaFC device. With the procedure, the accuracy is even slightly lower (83,03%) compared to original accuracy based on the score without normalization factors (84,24%). As the segmentation procedure provides only small correction factors to the final score and is not a part of the spectra classification algorithm, the detailed discussion on segmentation of skin lesions is beyond the scope of this paper. However, a comment should be made here on the reasons for choosing Li thresholding. Since every image in the PMCF study was taken with the same geometry and illumination conditions and the images were de facto artifact-free (no hair, oil, ruler markings, etc.), segmentation of pigmented skin areas was reduced to finding the optimal global thresholding value. The Li thresholding algorithm was found superior over other thresholding approaches tested (including Otsu, Yen, Isodata, and Niblack) as it works well with low-contrast areas in the images. Supervised learning algorithms could not be employed due to an insufficient amount of annotated data. Nevertheless, more systematic research is needed to find an optimal segmentation algorithm, most probably utilizing more advanced techniques, such as convolutional neural networks [33,34].

The DermaFC score provides diagnostic support for a dermatologist. For a score value higher than 28, excision is recommended. In practice, the method’s high specificity implies any lesion identified by the DermaFC device as melanoma should be excised. Because the final score provided by the device is a linear function of the number of spectra classified by the SVM algorithm as a characteristic for melanocytic malignancy, the value of the score can be linked to the probability of the lesion of being malignant melanoma. In other words, to further reduce the risk of missing melanoma, in cases where the scan revealed a score lower than 28, but still a high value, the lesion under investigation should be treated with caution and the patient potentially involved in a follow-up procedure. 

It is important to note the not-trivial problem of the sample labelling. Although the ground truth in this PMCF study was improved by examining each lesion by three independent dermatopathologists, misclassification remains possible. In particular, the nevi with high order of dysplasia might be incorrectly identified as melanoma by a pathologist. The melanoma overdiagnosis has been recently the subject of intense debate, also in the context of lesions that are morphologically malignant but biologically benign (e.g., some Spitz’s nevi, and nevi in “special sites,” such as acral skin or genitalia) [35,36,37].

Finally, a limitation of this PMCF-study is the relatively small number of samples involved. Future prospective studies on a larger number of lesions would be needed for a more precise estimation of the diagnostic accuracy of dermatofluoroscopy. 

In conclusion, early excision remains the only strategy to reduce mortality associated with melanoma, but unnecessary excision of benign lesions increases morbidity and raises healthcare costs associated with melanoma screening. The dermatofluoroscopy provides objective, spatially-resolved information about lesion malignancy as the fluorescence spectra from melanin in nevomelanocytes and healthy skin have different characteristics compared to malignant melanoma. After the scan with the DermaFC device is completed, the operator receives the dermoscopic-quality image with marked microareas of malignant degeneration, as well as the score, both supporting the physician in accurate melanoma detection. This PMCF study on 214 PSLs reveals the great potential of dermatofluoroscopy in helping specialized and non-specialized clinical settings to reduce the number “needed to excise” (NNE), calculated as the number of melanocytic lesions excised for every confirmed melanoma, without increasing the risk of overseeing melanoma. In the validation set, from 165 PSLs excised as lesions suspected of being malignant melanoma, only 24 were truly positive as confirmed by pathology (NNE = 6.875). The remaining 141 were benign lesions, of which 114 were correctly classified as non-melanoma with dermatofluoroscopy. Using DermaFC device, the number of superfluous excisions would have been reduced by 83% compared to clinical examination, whereas only one melanoma from the validation set of 165 lesions was missed (two of three pathologists agreed on this diagnosis), with the score of 27, very close to the cut-off value. Incorporation of this diagnostic technique in clinical practice will likely improve melanoma detection accuracy, mostly by reducing the excision rate of benign lesions.

## Figures and Tables

**Table 1:** Diagnostic accuracy of the dermatofluoroscopy validated on 165 lesions with and without region of interest (ROI)-normalization.

	Histopathology (Gold Standard)	Derma FC Diagnosis without Normalization of ROI	Derma FC Diagnosis with Normalization of ROI
melanoma	24	22	23
non-melanoma	141	117	114
sensitivity/specificity DermaFC	-	91.7%/83.0%	95.8%/80.9%

**Table 2:** Summary of false positive results.

Histopathology Diagnosis	*n*
Unequivocal (3 nMM)	22
Equivocal (1 MM, 2 nMM)	5
Nevus	11
Dysplastic Nevus	12
Other (e.g., pigmented BCC)	4
